# Therapy drives genomic evolution in metastatic cancer

**DOI:** 10.18632/oncotarget.28379

**Published:** 2023-03-21

**Authors:** Ditte S. Christensen, Nicolai J. Birkbak

**Keywords:** cancer evolution, metastatic cancer mutations, treatment-induced mutations, GENIE

Development of metastatic cancer is generally the lethal step in cancer. Yet, while considerable work has been performed over the past decade to unravel the genomic architecture of primary tumors, comparatively little effort has focused on understanding the genomic architecture of metastatic tumors. It is generally accepted that development of cancer is a slow process, likely spanning decades during which the developing neoplastic cells sequentially acquire genomic alterations that will eventually give rise to the primary tumor [1]. While cancer remains localized to a primary tumor, it is often susceptible to curative intervention. This contrasts starkly with metastatic cancer, which is essentially incurable once it has seeded to distant sites. This contrast suggests that the ability to metastasize is not inherent to cancer cells, but must be acquired during cancer development. The metastatic process itself involves multiple steps, including local invasion, intravasation, survival in circulation, extravasation, and colonization to distant tissues [2, 3]. How the ability to perform these multiple independent steps is acquired by cancer cells remains a mystery. Extensive genomic characterization of primary tumors has revealed likely genomic drivers of cancer, termed cancer driver mutations [4, 5]. Cancer driver mutations are defined as genomic alterations, mostly point mutations, indels, translocations, deletions or amplifications, which improve cancer cell fitness and thereby drive the development and/or progression of cancer. Analysis of multiple samples from individual tumors have revealed that most primary tumors are highly heterogeneous once they reach a size that enables detection [6], with multiple clones co-existing within the same primary tumor, each harboring slightly different mutational content, but all originating from the same common ancestor that initiated the carcinogenic process. Conversely, recent phylogenetic analysis of multiple cancer types has revealed that metastatic tumors are more likely to be of monoclonal origin [7], supporting a dissemination model that involves clonal bottlenecking, where metastatic potential is acquired by a single subclone, which seeds all metastatic tumors.

Considering a monoclonal dissemination process together with the evidence that primary cancer is commonly curable by surgery, while metastatic cancer is not, it is plausible that certain driver mutations are required for the development of metastatic potential, essentially acting as gatekeepers. Recently, two studies on metastatic cancer were performed by the Hartwig Medical Foundation (HMF), where the authors analyzed whole genome sequence data from 2520 metastatic tumors [8], and subsequently performed a paired analysis of serial samples from 250 patients [9]. Here, the authors reported an increase in overall mutation burden and chromosomal instability, consistent with the hypothesis of clonal bottleneck [7]. However, they found no metastasis-specific driver mutations, and they observed a 99% concordance between serial samples (time-separated primary-metastasis or metastasis-metastasis). These data are consistent with an older study from 2017, where Robinson and colleagues [10] reported an increased mutation burden, along with global dysregulation of gene transcription, on a set of 500 metastatic tumors. More recently, a study using panel-based tumor sequencing of 25,000 cancer patients treated at Memorial Sloan-Kettering observed increased mutation burden and chromosomal instability in metastatic tumors, but while they observed an intriguing association between specific drivers and seeding to specific metastatic sites, little evidence was found of individual mutations driving the metastatic process itself [11]. Thus, to this day, the concept of gatekeeper mutations remains a hypothesis.

Given the paradox that metastatic disease appears to occur as a binary event, clearly separating curable, localized cancer from disseminated, terminal cancer, we recently endeavored to explore the concept of gatekeeper genomic events in greater detail by comparing primary to metastatic tumors on a large scale [12]. The AACR Genomics Evidence Neoplasia Information Exchange (GENIE [13]) project includes panel-based genomic data from more than 100,000 tumors, making it the largest publicly available collection of cancer genomic data. The GENIE dataset includes tumors from a range of hospital sites, analyzed using different gene panels. In order to make the dataset comparable across sites, we defined a core set of 174 cancer genes, which had been analyzed in more than 40,000 individual tumors, including 24,333 primary tumors and 16,546 metastatic tumors. While this gene set is of limited scope relative to the full size of the human genome, these genes harbored more than 50% of the cancer driver mutations found in 2520 metastatic tumors analyzed by the HMF study [8], making it valid for the analysis of cancer driver mutations across the largest cohort of samples analyzed to date. Consistent with previous work suggesting clonal bottlenecking is common during metastatic dissemination [9–11, 14], we observed an increase in tumor mutation burden and chromosomal instability in metastatic tumors (Figure 1A). However, individual driver mutations showed limited variation between primary and metastatic disease. This observation remained true when mutations were summarized to pathway-level. We did observe a general trend towards an increase in frequency in metastatic tumors of events generally associated with tumor aggressiveness, such as TP53 mutations, MYC amplifications, and deletion of CDKN2A and PTEN. However, while the observed increase varied between cancer types, it was generally less than 10% relative to primary tumors (Figure 1B). When we analyzed individual variants, we found that a subset of mutations was clearly enriched in metastatic tumors. These were point mutations to ESR1, AR, EGFR and KIT, found strongly enriched in breast, prostate, non-small cell lung cancer and gastrointestinal tumors. All variants were previously reported as specifically conferring treatment resistance to anti-estrogens, anti-androgens, anti-EGFR and imatinib. Taken together, this suggests that a general model of metastatic cancer dissemination may take the form of a bottleneck event where a highly fit clone from a heterogeneous primary tumor successfully seeds to distant sites. Absent therapy, the selective pressures remain similar in primary and metastatic tumors. However, once subjected to anti-cancer therapy, strong selective pressure drives the acquisition of driver mutations associated with therapy resistance, private to individual metastatic tumors (Figure 1C).

**Figure 1:**
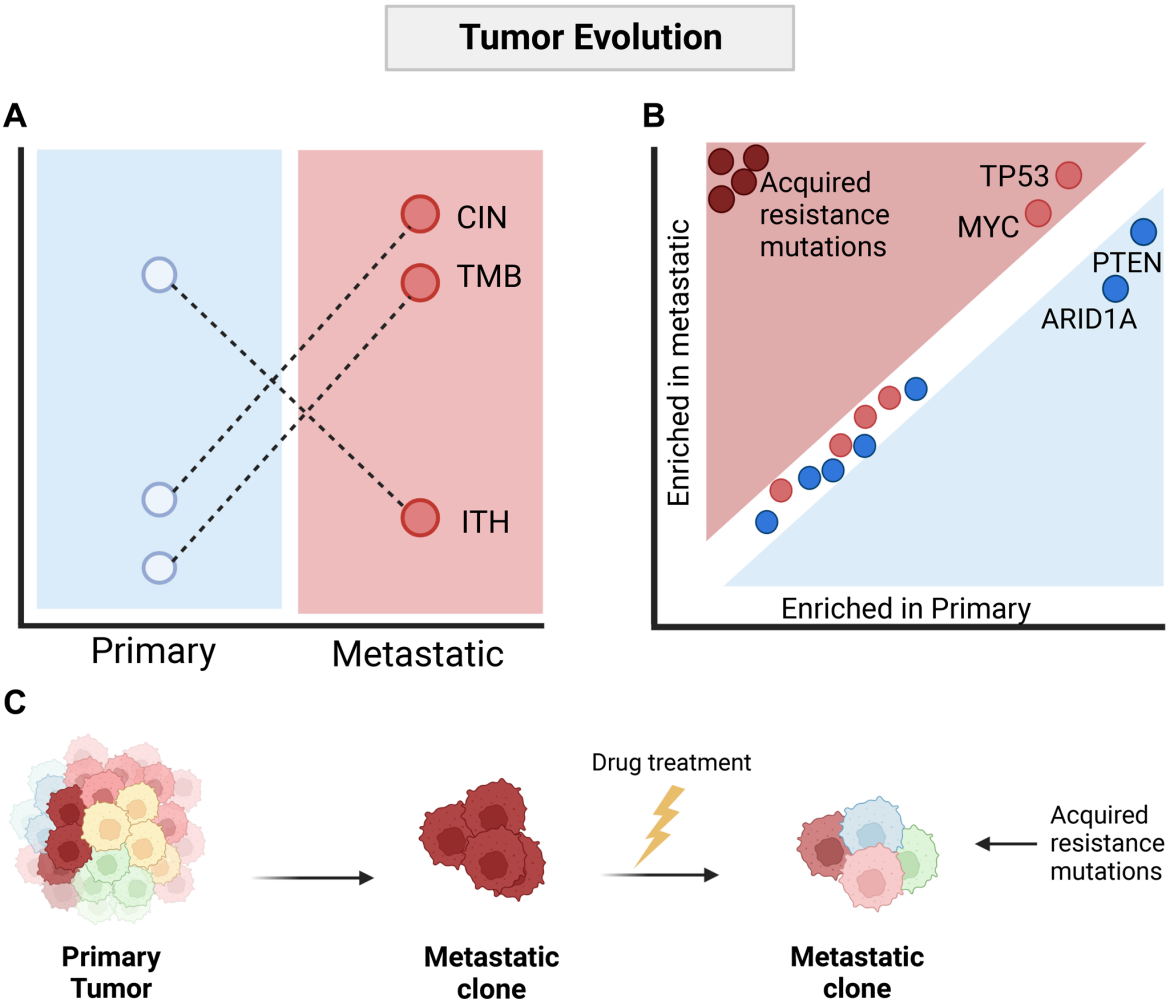
Evolution of the metastatic cancer genome is dominated by clonal bottlenecking and anti-cancer treatment. (**A**) Metastatic tumors are commonly observed as predominantly clonal, and with a general increase in tumor mutation burden and chromosomal instability relative to primary tumors. Abbreviations: TMB: Tumor mutation burden; CIN: Chromosomal instability; ITH: Intratumor heterogeneity. (**B**) Comparison of driver mutations in primary and metastatic tumors shows high consistency in the driver landscape, with only minor increases in aggressive cancer traits such as TP53 mutations and MYC amplifications observed in metastatic cancer. Only mutations associated with resistance, likely induced by anti-cancer therapy, are strongly enriched in metastatic cancer. (**C**) General model of metastatic cancer evolution. Created with http://BioRender.com.

When we consider our own recent work [12, 14] and that of others [1, 8–11], there is limited evidence for the existence of specific gatekeeper mutations. Rather, the strong correlation observed between genomic events in primary and metastatic tumors indicate that in the absence of treatment, the evolutionary pressures are similar and from a genomic standpoint mostly focused on acquisition of aggressive cancer traits through inactivation of tumor suppressor genes such as TP53, and activation of proliferative genes such as MYC. While it is possible that genomic events outside of known cancer genes or in non-coding regions may selectively drive the metastatic process, these data suggest that the process of developing metastatic potential is driven less by individual events. Indeed, it may be that a primary driver of metastatic cancer is to be found outside the cancer cells themselves, potentially through inflammation in the tumor-immune microenvironment or through interaction with a declining host immune system which may enable immune escape and sudden systemic dissemination by a highly proliferative primary tumor clone. In the long run, fully understanding the key steps that drive the development of metastatic potential is critical to improve cancer outcome. It will be exciting to further explore these questions as more data becomes available on metastatic cancers, particularly with paired primary and metastatic tumor samples with sequential biopsies to facilitate the analysis of dynamic tumor evolution over time, rather than through static snapshots provided by samples obtained at a single time point.

## References

[1] Reiter JG , et al. Nat Rev Cancer. 2019; 19:639–50. 10.1038/s41568-019-0185-x. 31455892 PMC6816333

[2] Hanahan D , et al. Cell. 2011; 144:646–74. 10.1016/j.cell.2011.02.013. 21376230

[3] Chaffer CL , et al. Science. 2011; 331:1559–64. 10.1126/science.1203543. 21436443

[4] Bailey MH , et al. Cell. 2018; 173:371–85.e18. 10.1016/j.cell.2018.02.060. 29625053 PMC6029450

[5] Lawrence MS , et al. Nature. 2014; 505:495–501. 10.1038/nature12912. 24390350 PMC4048962

[6] McGranahan N , et al. Cell. 2017; 168:613–28. 10.1016/j.cell.2017.01.018. 28187284

[7] Birkbak NJ , et al. Cancer Cell. 2020; 37:8–19. 10.1016/j.ccell.2019.12.004. 31935374

[8] Priestley P , et al. Nature. 2019; 575:210–16. 10.1038/s41586-019-1689-y. 31645765 PMC6872491

[9] van de Haar J , et al. Nat Med. 2021; 27:1553–63. 10.1038/s41591-021-01448-w. 34373653

[10] Robinson DR , et al. Nature. 2017; 548:297–303. 10.1038/nature23306. 28783718 PMC5995337

[11] Nguyen B , et al. Cell. 2022; 185:563–75.e11. 10.1016/j.cell.2022.01.003. 35120664 PMC9147702

[12] Christensen DS , et al. Cancer Res. 2022; 82:2918–27. 10.1158/0008-5472.CAN-22-0562. 35731928

[13] AACR Project GENIE Consortium. Cancer Discov. 2017; 7:818–31. 10.1158/2159-8290.CD-17-0151. 28572459 PMC5611790

[14] Ahrenfeldt J , et al. Cancers (Basel). 2022; 14:5817. 10.3390/cancers14235817. 36497297 PMC9739002

